# No Prophylactic Antibiotic Use for Young Children’s Intussusception with Low-risk Infection after Successful Air Enema Reduction

**DOI:** 10.1038/s41598-018-24415-x

**Published:** 2018-04-17

**Authors:** Yuan Zhang, Wen Zou, Lemeng Ren, Yinghui Zhang, Zhaohui Sun, Huandi Liu, Qian Liu, Chunfeng Si, Hongying Jia

**Affiliations:** 1 Center of Evidence-based Medicine, Second Hospital of Shandong University, Jinan, China; 2 School of Public Health, Shandong University, Jinan, China; 3 Department of Pharmacy, Second Hospital of Shandong University, Jinan, China; 4 School of Basic Medical Sciences, Lanzhou University, Lanzhou, China; 5 Department of Medical Records, Second Hospital of Shandong University, Jinan, China; 6 Department of Laboratory, Second Hospital of Shandong University, Jinan, China; 7 Information Center, Second Hospital of Shandong University, Jinan, China; 8 Department of Paediatrics, Second Hospital of Shandong University, Jinan, China

## Abstract

The Chinese government has issued the policy of promulgating the clinical use of antibacterial drugs since 2011. Prophylactic antibiotic use is a challenging problem among young children with intussusception after successful air enema reduction. There were limited data regarding the clinical value of prophylactic antibiotics for intussusception with low-risk infections. A retrospective non-randomized comparative study was conducted among 188 young children with intussusception after successful air enema reduction between January 1, 2011 and December 30, 2013. Among these children, 51 received prophylactic antibiotics and 137 did not receive antibiotics. Our results showed that there were no significant differences in age, gender, weight, admission period, reduction interval, axillary temperature, leukocytes, neutrophils, lymphocytes, monocytes, mesenteric lymph nodes and complications between groups (*P* > 0.05). The national policy had significantly improved clinical use of antibiotic for young children with low-risk intussusception (*OR* < 0.001, *P* < 0.001). Inpatients days were longer for children used antibiotics than those who did not (median, 27.0 hours vs 21.0 hours, *P* = 0.003). Prophylactic antibiotics appeared to be of little value after the successful air enema reduction of intussusception in young children with low-risk infection. Policy intervention is effective for antibiotic use in China.

## Introduction

Intussusception is the most common cause of intestinal obstruction in young children under 36 months of age. Approximately 60 percent of affected children are younger than one year old, and 80 to 90 percent are younger than two years old^1^. Intussusception occurs when a segment of the bowel (the intussusceptum) telescopes into a more distal bowel (the intussuscipiens), resulting in venous congestion and bowel wall edema^2^. As a non-operative technique, air enema reduction is quick and clean with a high reduction rate (73–95%) and less radiation exposure^3^. The main risk is bowel perforation, which occurs in 1% or fewer patients^4^. To prevent the intra-abdominal infection, the Infectious Diseases Society of America recommended the specific antimicrobial therapy based on the origin, severity and safety of the antimicrobial agents for paediatric patients^5^. However, there is no worldwide consensus on whether antibiotics should be applied prophylactically for patients with low-risk intussusception before and after enema reduction^6–8^. In China, antibiotics were prescribed to children with intussusception in almost all hospitals since there is no evidence-based guideline. And the prescription involved different kinds of antibiotics^9^. However, the value of prophylactic practice has not been evaluated. Therefore, we conducted this study to explore whether the prophylactic application of antibiotics had clinical significance for patients with low-risk intussusception.

## Results

### Characteristics of participants before reduction

**Figure 1:**
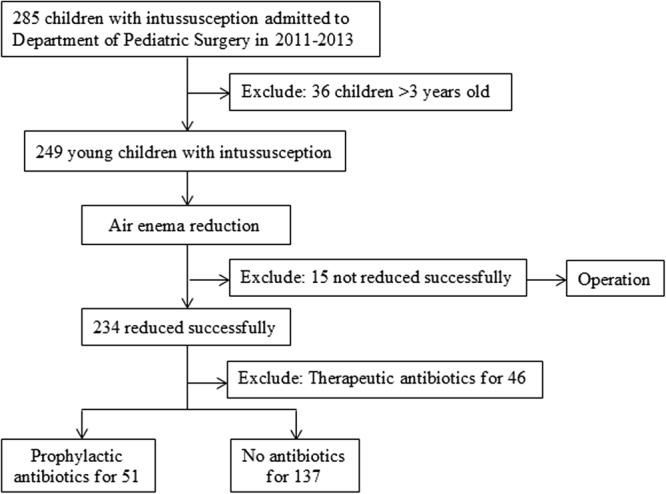
Procedure of recruiting young children for antibiotic use analysis.

As shown in Fig. 1, a total of 188 children less than three years old with intussusception were recruited, including 51 (27.1%) with prophylactic antibiotics and 137 (72.9%) without antibiotics. No significant differences were observed between two groups in age, gender, weight, admission period, axillary temperature, leukocytes, neutrophils, lymphocytes, monocytes, mesenteric lymph nodes and complications (*P* > 0.05). Both the length and diameter of masses corresponded to the ultrasonographic findings in paediatric cases with intussusception. The two groups were comparable (Table 1).

**Table 1:** Comparison of physiological characteristics among young children with intussusception before air enema reduction (mean ± SD, n, %).

		Prophylactic antibiotics (n = 51)	No antibiotics (n = 137)	*t/χ* ^2^	*P*
Age (months)		18.9 ± 10.2	19.7 ± 11.4	0.422	0.673
Weight (kg)		11.6 ± 2.8	12.0 ± 2.9	0.971	0.333
Length of mass (mm)		47.5 ± 9.4	42.2 ± 12.4	−3.118	0.007
Diameter of mass (mm)		25.9 ± 5.1	23.8 ± 5.8	−2.254	0.025
Gender	male	32 (62.8)	91 (66.4)	0.222	0.637
	female	19 (37.3)	46(33.6)		
Admission period	0:00–7:59	7 (13.7)	13 (9.5)	0.879	0.644
	8:00–16:59	25 (49.0)	75 (54.7)		
	17:00–23:59	19 (37.3)	49 (35.8)		
Temperature (°C)	normal	40 (78.4)	99 (72.3)	0.734	0.392
	>37.4	11 (21.6)	38 (27.7)		
Leukocytes (10^9^/L)	normal	34 (66.7)	96 (70.1)	0.202	0.653
	>9.5	17 (33.3)	41 (29.9)		
Neutrophils (10^9^/L)	normal	44 (86.3)	102 (74.5)	2.994	0.084
	>6.3	7 (13.7)	35 (25.6)		
Lymphocytes (10^9^/L)	normal	23 (45.1)	68 (49.6)	0.306	0.580
	>3.2	28 (54.9)	69 (50.4)		
Monocytes (10^9^/L)	normal	27 (52.9)	56 (40.9)	2.194	0.139
	>0.6	24 (47.1)	81 (59.1)		
Mesenteric lymph	normal	23 (45.1)	64 (46.7)	1.418	0.492
	hyperplasia	27 (52.9)	65 (47.5)		
	lymphadenitis	1 (2.1)	8 (5.8)		
Complications	none	51 (100.0)	131 (95.6)	2.307	0.129
	have	0 (0.0)	6 (4.4)		

### Impact of policy intervention to antibiotic use among young children with intussusception

**Figure 2:**
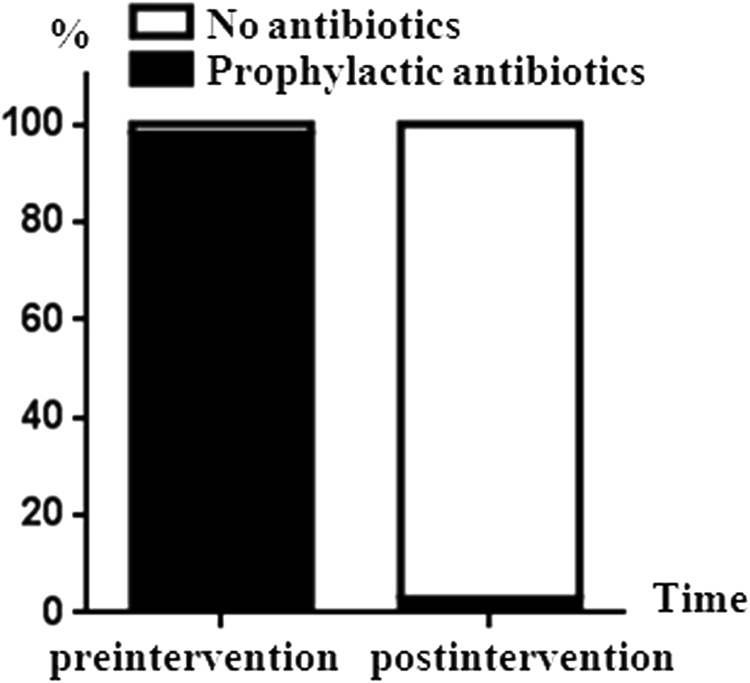
The change in the proportion of prophylactic antibiotics in the preintervention and postintervention periods.

In the preintervention period, the proportion of prophylactic antibiotics usage was as high as 97.9% (47/48). In the postintervention period, this proportion sharply decreased to 2.9% (4/140). The national policy had significantly improved clinical use of antibiotics (χ^2^ = 163.391, *P* < 0.001) (Fig. 2). Variables that had difference between two groups in univariate analysis were included into logistic regression model (Table 2). It showed that policy intervention was an independent protective factor against antibiotic use after successful air enema reduction (OR < 0.001, *P* < 0.001).

**Table 2:** Logistic regression analysis of antibiotic use among young children with intussusception.

	Estimate	SE	χ^2^	OR	*P*
Length of mass (mm)	0.088	0.059	2.206	1.092	0.137
Diameter of mass (mm)	0.039	0.102	0.148	1.040	0.700
Policy intervention	−8.139	1.381	34.715	<0.001	<***0.001***

### Comparison of prognosis and hospital costs in children based on antibiotic administration

All the participants in two groups were cured. However, children who received prophylactic antibiotics had longer inpatients days than those who did not (median, 27.0 hours vs 21.0 hours, *P* = 0.003).

The median inpatient cost was CNY 1201.7 and 1120.6 in the prophylactic antibiotics and no antibiotics groups, respectively, without significant difference (*P* = 0.376). The procedure cost and material cost (including infusion tube and other medical consumables) was higher in the prophylactic antibiotics group than that in the no antibiotics group (*P* < 0.001). The median antibiotic cost was up to CNY 205.0 in the prophylactic antibiotics group. The costs of imaging, laboratory test, bed and nursing care had no differences in two groups (*P* > 0.05). The results are shown in Table 3.

**Table 3:** Comparisons of costs (CNY) among children with intussusception based on antibiotic administration (median, inter-quartile range, n, %).

	Prophylactic antibiotics (n = 51)	No antibiotics (n = 137)	Z*/χ*^2^	*P*
Short-term efficacy (cured)	51 (100.0)	137 (100.0)	0.000	1.000
Inpatients days (hours)	27.0 (20.5–37.5)	21.0 (16.3–28.8)	−2.955	***0.003***
Total inpatient cost	1201.7 (960.1, 1573.3)	1120.6 (982.8, 1428.3)	−0.885	0.376
Imaging cost	330.0 (330.0, 330.0)	330.0 (330.0, 330.0)	−1.815	0.070
Laboratory cost	42.0 (20.0, 45.0)	25.0 (0.0, 50.0)	−0.244	0.807
Procedure cost	232.0 (219.0, 245.0)	220.0 (215.0, 227.0)	−4.278	<***0.001***
Antibiotics cost	205.0 (140.0, 333.0)	0.0 (0.0, 0.0)	−13.451	<***0.001***
Bed cost	50.0 (40.0, 80.0)	50.0 (40.0, 60.0)	−0.003	0.998
Nursing cost	9.0 (9.0, 18.0)	9.0 (9.0, 18.0)	−0.933	0.351
Materials cost	160.7 (139.4, 194.9)	132.3 (96.9, 152.9)	−4.981	<***0.001***

## Discussion

Intussusception is one of the leading causes of intestinal obstruction in the paediatric population^6^. Abdominal pain is the most common complaint. Ileocolic intussusception represents 90% of all intussusceptions with multifactorial and largely unknown etiology^10^. Intussusception tends to be complicated or secondary to infection, and complications such as fever, diarrhea, bloody stools, ischemia colitis, perforation and intestinal necrosis can occur and even result in bowel resection and death if left untreated^10^. Therefore, routine antibiotic administration before enema reduction is often advocated for intussusception. The USA Pediatric Surgery book states that broad-spectrum antibiotics should be given as for any other situation in which the vascular supply of the bowel may be jeopardized^6^. Similarly, the Children’s Hospital of Illinois in USA recommended that before any treatment of intussusception, 1 dose of cefoxitin (40 mg/kg) should be preferred. Although perforation is rare, this treatment provides antibiotic coverage against potential complications. In patients with gross peritonitis, coverage with broad-spectrum antibiotics is necessary, and the length of treatment should be determined once the degree of contamination is determined^11^.

Intussusception is also an important cause of morbidity in children. Thus, a timely, early diagnosis and a rapid non-operative intervention is vital to successful outcomes^12^. In China, paediatricians generally believe that antibiotics should be prescribed immediately after air enema reduction to address possible infectious complications. However, this practive has led to antibiotics abuse for intussusception among children with low or no infectious risk. In 2011, the Chinese government launched a special campaign against irrational use of antibiotic and promulgated the Administrative Measures for the Clinical Use of Antibacterial Drugs^13^. According to the guideline, prophylactic antimicrobial agents should be used for high-risk patients without bacterial infection but exposing to pathogenic bacteria, or those who were treated for primary diseases that may increase infectious risk^14^.

Children in this study were at low risk of infection. We did not observe any differences in age, gender, weight, admission period, axillary temperature, white blood cells, neutrophils, lymphocytes, monocytes and mesenteric lymph nodes with the presence or absence of antibiotic use after reduction. The length and diameter of masses were consistent with Ayaz UY’s findings^15^. Although the ultrasonographic results were significantly different in our two groups, they were not risk factors for antibiotic use in logistic regression analysis. Hyperplasia and lymphadenitis of the mesenteric lymph nodes were induced usually by viral or bacterial infections in most children who had intussusception^16^. Sixty-five children with hyperplasia and eight with lymphadenitis were cured without antibiotic.

The discovery of antibiotics in the 1930s fundamentally transformed the way physicians take care for patients, shifting their approach from a focus on diagnosis without means to intervene to a treatment-focused approach that saves lives^17^. Antimicrobial agents have made a major breakthrough in the promotion and protection of children’s health, at the same time, they cause obvious side effects, such as rash, diarrhea and clostridium infection. Additionally, the increasing use of antibiotics could eliminate the weaker bacteria and select the stronger ones; in other words, it breeds superbacteria that are resistant to multiple antibiotics^18^. Antimicrobial agents can also alter the normal gastrointestinal flora and be associated with autoimmune diseases, allergies, asthma, obesity and other diseases^19^. Fortunately, no short-term adverse antibiotic reactions were observed in our study.

Regarding evidence-based medicine for paediatricians, one study in Canada revealed that it appeared of little value to apply antibiotics before enema reduction of intussusception^7^. Japanese guidelines for the management of intussusception in children (Edition 2011) also stated that the routine administration of antimicrobial drugs after enema reduction was not necessary. Serial blood culture studies both pre-enema and post-enema did not justify the routine administration of antibiotics after enema reduction. The risk of bacteremia from enteric pathogens following air enema for the reduction of intussusception in children appears to be low^20^. Antimicrobial therapy was justified only in the presence of underlying sepsis. In this study, the significant reversed proportion between prophylaxis and no-use antibiotics was due to the policy intervention in China. In other words, policy intervention is effective in improving the normative use of antibacterial drugs among young children with intussusception. We also found that children who received prophylactic antibiotics had longer inpatient days than children who did not (median, 27.0 hours vs 21.0 hours, *P* = 0.003). The median antibiotic cost was up to CNY 205.0. The procedure cost and other costs were higher in the prophylactic antibiotics group compared to the no antibiotic group. However, there was no difference between two groups of children regarding treatment outcomes.

Based on our findings, we suggest that it’s unnecessary to use prophylactic antibiotic for young children’s intussusception with low-risk infection. After successful reduction, children with intussusception should be continuously observed for at least 24 h to prevent the appearance of abnormal conditions such as crying, fever, defecation (with or without diarrhea and bloody stools) and abdominal signs. Children complicated with infectious diseases, such as upper respiratory tract infection, bronchitis, bronchopneumonia, enteritis, perforation and even intestinal necrosis, should be monitored.

If our study was a multi-central, prospective and randomized-control trial with a larger sample size, the results would be more powerful. Despite these limitations, we still obtained some convincing results about evidence-based pharmacy for medication guide in young children with low-risk intussusception. We also observed the positive influence of the national policy in the improvement of antibiotic use in China.

## Methods

### Selection of Children with Intussusception

After approval from the institutional review board of Second Hospital of Shandong University, a retrospective and non-randomized comparative study (NRCS) was conducted among intussusception patients treated at the department of paediatric surgery between January 1, 2011 and December 30, 2013. We focused on the diagnosis code of K56.1 (International Classification of Diseases, 10^th^ revision). Informed consent was signed by the parents of each child before the air enema was administered. Children who were less than three years old, diagnosed as first episode of intussusception and received reduction with air enema were included, while those who received surgery due to unsuccessful air enema or used antibiotic use for therapy intention were excluded. We divided these patients into “Prophylactic Antibiotics Group” and “No Antibiotics Group”. Patients’ characteristics, policy intervention, prognosis and costs were compared between two groups.

The quality of this NRCS was assessed using the Methodological Index for Nonrandomized Studies (MINORS)^21^. This index contains 12 items that are scored as 0 (not reported), 1 (reported but inadequate), or 2 (reported and adequate). The ideal global score was 24, and our study had a score of 19.

### Evaluation of patients’ characteristics

Data were collected from the Department of Medical Records, Laboratory, Pediatric Surgery, Pharmacy and Information, including demographics, weight, admission period, reduction interval, mass size, axillary temperature, blood test results, mesenteric lymph nodes, complications and short-term efficacy. Blood test indicators included leukocytes, neutrophils, lymphocytes and monocytes. Complications during the admission were mainly the upper respiratory tract infection and enteritis. The criteria of cure were successful reduction and discharge from hospital without intussusception relapse.

### Policy and Economic analysis

Our hospital responded to the national policy to strengthen the management of antibiotic prescriptions and promoted the reasonable clinical use of antibacterial drugs from October 31, 2011. Therefore, the “policy intervention” was divided into two periods, which were preintervention (January 1, 2011 to October 31, 2011) and postintervention periods (November 1, 2011 to December 31, 2013). Economic analysis focused on the analysis of hospital expenses. Total expenses included costs of imaging, laboratory, procedure, medicine, bed, nursing and materials.

### Statistical analysis

Data was analyzed using SAS version 9.4 (SAS Institute, Cary, NC, USA). Normal distribution data were expressed as mean ± standard deviation (SD), and non-normal distribution data were represented by median and inter-quartile range. Differences between groups were compared using *t* test for continuous variables with normal distribution, Mann-Whitney *U* test for non-normal variables and Chi-square test for categorical variables. Risk factors of antibiotic use were analyzed by employing logistic regression model. A *P* value less than 0.05 was considered statistically significant.
